# *COL5A1* rs13946 Polymorphism and Anterior Cruciate Ligament Injury: Systematic Review and Meta-Analysis

**DOI:** 10.3390/ijms26136340

**Published:** 2025-06-30

**Authors:** Zhuo Sun, Paweł Cięszczyk, Aleksandra Bojarczuk

**Affiliations:** 1 College of Physical Education, Chengdu Normal University, Chengdu 611130, China; 2 Faculty of Physical Culture, Gdansk University of Physical Education and Sport, 80-336 Gdansk, Poland; pawel.cieszczyk@awf.gda.pl (P.C.); aleksandra.bojarczuk@awf.gda.pl (A.B.)

**Keywords:** genetic risk, SNP, sport injury, collagen, ACL

## Abstract

Anterior cruciate ligament (ACL) injury (ACLI) is a prevalent sports injury. Genetic factors play a crucial role in determining the risk of ACLI. This systematic review aimed to identify the association between the *COL5A1* rs13946 polymorphism and susceptibility to ACLI. Methods: Searches were performed in PubMed Central, Web of Science, EBSCOhost, Scopus, and CNKI. The Newcastle–Ottawa Scale (NOS) was used to assess potential bias, and data from the included studies were analyzed using RevMan 5.4. The odds ratio (OR) and 95% confidence intervals (95% CI) were calculated to determine the strength of the association between *COL5A1* rs13946 and the risk of anterior cruciate ligament injury. A *p* value < 0.05 was considered statistically significant. Seven studies met the inclusion criteria for screening the association between *COL5A1* rs13946 and ACL injury and were included in this meta-analysis. The meta-analysis revealed no significant heterogeneity across five genetic models. Statistically significant findings were observed in the recessive (OR = 1.29, 95% CI [1.06, 1.58], *p* = 0.01) and allele models (OR = 0.85, 95% CI [0.73, 1.00], *p* = 0.04). The TT genotype or T allele of rs13946 showed a distinct susceptibility to ACLI under the recessive model, particularly in Caucasians. This study supports the association between *COL5A1* rs13946 and the risk of ACLI, particularly in Caucasians. More specifically, the C/- genotype of rs13946 provides protection against ACLI in Caucasians. Further research with larger sample sizes and well-balanced gender-specific cohorts is necessary to validate this association and strengthen our findings.

## 1. Introduction

Anterior cruciate ligament injury (ACLI) is one of the most common musculoskeletal injuries in sports, with over 120,000 cases annually in the U.S. alone [1], and approximately 70% occurring via non-contact mechanisms [2]. While mechanical, hormonal, and environmental factors have been implicated [3,4,5], not all individuals exposed to the same risk factors develop ACLI, suggesting a genetic component [6,7].

Type V collagen, encoded by the *COL5A1* gene on chromosome 9, plays a critical role in the structural integrity of ligaments [8,9,10,11]. Genetic variations within *COL5A1*, particularly single-nucleotide polymorphisms (SNPs), have been associated with soft tissue injuries, including ACLI [12,13], Achilles tendon injuries [14,15], and rotator cuff injuries [16,17]. Among these, the rs13946 polymorphism within the 3′-untranslated region (UTR) of *COL5A1* has emerged as a potential risk factor. However, results from individual studies have been inconsistent. For example, Posthumus et al. (2009) found no significant association between the *COL5A1* rs13946 (DpnII) polymorphism and ACLI in either sex [13], while Lulińska-Kuklik et al. (2018) reported that the C-C haplotype of rs12722–rs13946 in the *COL5A1* gene was significantly overrepresented in controls, suggesting a potential protective effect against anterior cruciate ligament (ACL) rupture in Polish male soccer players [18]. Some studies observed associations only in females [19]. For instance, Rodas et al. (2023) found a significant association between the rs13946 C/C genotype and ACL injury in elite female footballers, but not in males, indicating a potential female-specific genetic risk [19]. Some studies observed associations in specific ethnic groups, such as Italian athletes [20], while others, such as Zhao et al. (2020), reported no association in Chinese Han participants [21]. These discrepancies may stem from small sample sizes, ethnic heterogeneity, and differing study designs, making it difficult to draw firm conclusions. Although a genetic predisposition to ACLI is well supported, the specific contribution of *COL5A1* rs13946 remains inconclusive. In addition to the studies discussed above, several others have reported associations between *COL5A1* rs13946 and musculoskeletal soft tissue injuries (MSTIs), further supporting its potential relevance [18,22,23,24,25,26,27]. Despite previous studies exploring the *COL5A1* rs13946 polymorphism’s potential association with ACLI, significant gaps remain in the literature, particularly regarding resolving conflicting findings attributed to limited sample sizes and the diversity of populations examined. This meta-analysis aims to clarify the relationship between the *COL5A1* rs13946 polymorphism and susceptibility to ACLI by systematically reviewing and synthesizing data from seven independent studies covering a range of ethnicities and demographics [13,18,19,20,21,26,27]. Specifically, we analyzed associations using five genetic models—dominant, recessive, homozygote, heterozygote, and allelic—while also performing subgroup analyses by ethnicity and gender, thereby seeking to elucidate the role of *COL5A1* rs13946 across different populations and contribute valuable insights to the understanding of genetic risk factors associated with ACLI.

## 2. Materials and Methods

This study adhered to the guidelines outlined in the Preferred Reporting Items for Systematic Reviews and Meta-Analyses (PRISMA) statement for reporting [28]. This systematic review was registered in the PROSPERO database (https://www.crd.york.ac.uk/prospero/, date created on 1 April 2024, last accessed on 22 June 2025) under registration number CRD42024531011.

### 2.1. Search Strategy

Five electronic databases, PubMed Central, Web of Science, EBSCOhost, Scopus, and CNKI, were utilized for searching without language restrictions and time limitations. Different MeSH terms were filtered from each database before searching for the target studies. The following search strategy was used in SCOPUS: TITLE-ABS-KEY (“anterior cruciate ligament injury” OR “anterior cruciate ligament injuries” OR “ACL injury” OR “ACL injuries” OR “ACL tear” OR “ACL tears” OR “ACL rupture” OR “ACL ruptures”) AND TITLE-ABS-KEY (*COL5A1* OR rs13946 OR “*COL5A1* rs13946”). The Supplementary Files comprehensively document each database’s exact search dates and full search strategies.

### 2.2. Inclusion and Exclusion Criteria

We have implemented the methodology from our previous systematic review, with similar inclusion and exclusion criteria outlined in our prior study [29], but without language and time restrictions for this systematic review. The criteria included exploring genetic influences on human ACL injury using case–control, cohort, cross-sectional, or randomized controlled experiments. Studies that had been previously reviewed, such as animal studies, book chapters, letters, editorials, conference abstracts, or review articles, were excluded. Additionally, studies lacking full text were eliminated, as were those with fewer than ten participants. An extra specific criterion was introduced for this review to reduce bias: studies should have examined the association between the rs13946 polymorphism in the *COL5A1* gene and ACL injuries. The searches were executed between 10 March 2024 and 31 July 2024, adhering to the timeframe pre-specified in our PROSPERO protocol.

### 2.3. Selection Process and Data Extraction

Two authors independently searched the studies using the abovementioned search strategy. Related studies identified across five electronic databases were imported into the reference manager (EndNote X9.3.3 (Bld 13966)) to eliminate duplicates. Two authors conducted a preliminary screening by reading the titles and abstracts of the studies initially, and the full texts were examined if the abstracts did not provide the required information for the studies to be included.

Two authors reviewed the full texts to extract data from these qualified studies. The data were managed by using Microsoft Word and Excel (for Mac version 16.66 (22100900), 2022) in tabular format, which included the first author’s name, the year of publication, the ethnicity and gender of the subjects, the sample size and sample type, the genotype distribution of rs13946 in cases and controls, the diagnosis of cases from each study, and the Hardy–Weinberg equilibrium (HWE). For all studies included in the meta-analysis, the original authors assessed HWE for the control groups. A third senior reviewer was consulted in cases of discrepancy between the two authors.

### 2.4. Quality Assessment and Risk of Bias

The Newcastle–Ottawa Scale (NOS) was employed to evaluate potential bias in case–control, cohort, and cross-sectional studies as outlined by Stang in 2010 [30], which consists of three dimensions: selection (4 items), comparability of the case (3 items), and exposure (2 items). The NOS is a star rating system from zero to nine, with each item eligible for one star. The cumulative points are employed to assess the quality of studies, wherein scores of 7 or above indicate high quality, scores between 6 and 7 denote medium risk, and scores between 0 and 5 signify a high risk of bias. Two independent reviewers (*-S and **-B) evaluated the included studies based on the standards set by the star rating system. Subsequently, the NOS results were compared and discussed.

### 2.5. Statistical Analysis

The data from the included studies were analyzed using RevMan 5.4 (Cochrane Collaboration, London, UK). Although Egger’s regression test is widely recognized as an important statistical tool for detecting publication bias in meta-analyses, it could not be performed in the present study due to software limitations. Specifically, RevMan 5.4 does not support regression-based publication bias assessments. Nevertheless, visual inspection of the funnel plot did not reveal substantial asymmetry, suggesting a low risk of publication bias among the included studies [31]. ORs and 95% CIs were calculated to determine the strength of the association between *COL5A1* rs13946 and the risk of anterior cruciate ligament injury. A *p* value < 0.05 was considered statistically significant. This review concludes five genetic models. The pooled odds ratios (ORs) were calculated for the dominant model (TT + TC versus CC), recessive model (TT versus TC + CC), homozygote model (TT versus CC), heterozygote model (TC versus CC), and allelic comparison (T versus C). The subgroup was analyzed by sex and ethnicity to investigate further risks of ACLI. The heterogeneity was assessed using statistical chi-square-based Q and I2 values, with the random-effects model applied when *p* < 0.01 and I2 > 50%, indicating significant heterogeneity among the included studies; otherwise, a fixed-effect model was used.

To ensure consistency, we extracted detailed information about the genotyping methods reported in each study. Only those using validated and widely accepted genotyping techniques (e.g., PCR-RFLP, TaqMan, sequencing) were included. Studies with unclear or unreliable genotyping methods were excluded from the meta-analysis. As a result, all seven included studies met our criteria.

The sensitivity analyses excluded a single study that influenced the overall effect of all pooled data. The funnel plots were employed to reduce the potential publication bias.

## 3. Results

### 3.1. Search Results

**Figure 1:**
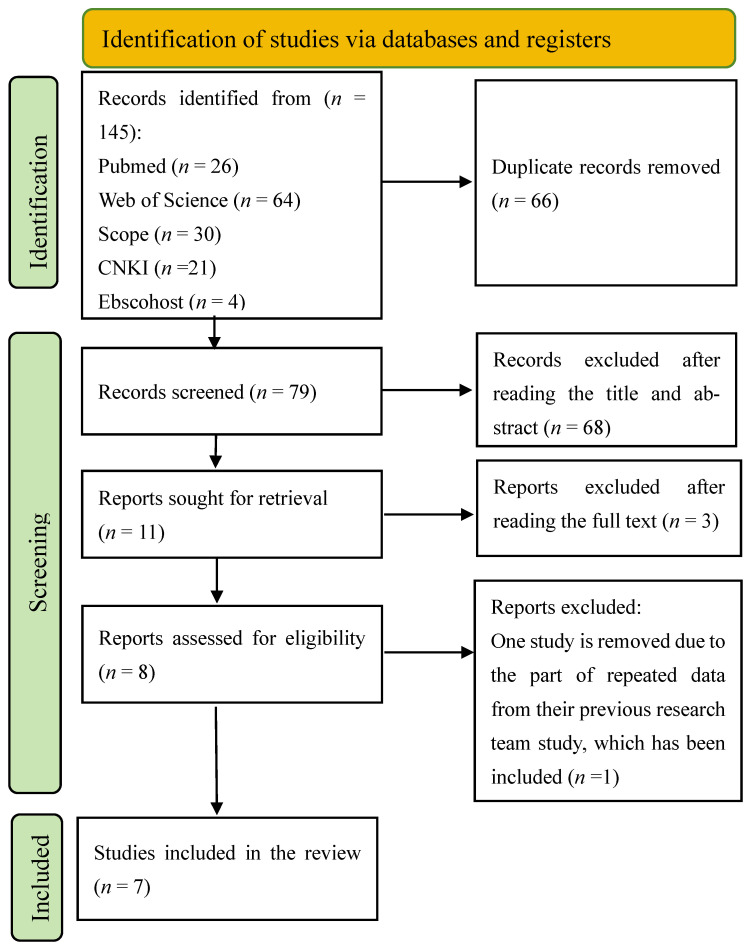
PRISMA flowchart showing the study-selection process.

The literature search yielded a total of 135 studies. After 56 duplicates were removed, 79 publications were screened. Furthermore, the titles and abstracts of the remaining 68 studies were independently reviewed by two reviewers. Subsequently, 11 full-text studies were assessed for detailed information, and 3 were excluded. One study was eliminated due to the presence of repeated data from a previous publication by the same research team, which had been incorporated into the present study. Consequently, the initial criteria were met by seven studies, including four case–control studies [13,18,20,27], two cohort studies [19,26], and one cross-sectional study [21]; Figure 1 depicts the selection process.

### 3.2. Study Characteristics

Seven studies contained a total of 715 cases and 1586 healthy controls. Four [13,18,27] studies investigated the association between rs13946 and ACL injury in the Caucasian population, three of these were conducted in Poland [13,18,27], and one was conducted in Italy [20]. Additionally, two studies [19,26] examined this association in a mixed-ethnicity population, with one study [19] focusing on a Norwegian-mixed-Finnish population in Spain, and another study [26] including a mix of Caucasian, African, and Latin American populations. Furthermore, one study specifically investigated the Asian population in China [21]. Table 1 displays the main characteristics, and Table 2 shows each study’s genotype frequencies of the rs13946 polymorphism, respectively.

### 3.3. Risk of Bias

The NOS was used to evaluate the quality of the study, with a rating greater than six indicating exceptional quality. Table 3 shows the NOS evaluation results of the included studies. One study [21] received six points, while the others were rated above six, indicating the exceptional quality of the included papers.

Six of the seven included studies reported HWE results, while one study [26] only stated that the sample followed HWE without providing detailed data. For this study, we manually calculated the HWE test to ensure consistency.

### 3.4. Meta-Analysis and Subgroup-Analysis

**Figure 2:**
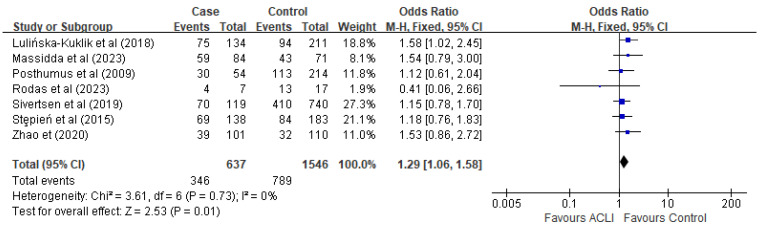
Forest plot of the recessive model (TT versus TC + CC) of *COL5A1* rs13946 for overall comparison. The blue points are the study’s OR. The blue horizontal line is 95% CI. References cited Lulińska-Kuklik et al., (2018) [18], Massidda et al., (2023), Posthumus et al., (2009) [13], Rodas et al., (2023) [19], Sivertsen et al., (2019) [26], Stepień-Słodkowska et al., (2015) [27], Zhao et al., (2020) [21]. RevMan 5.4 does not support the *p*-value in italics.

**Figure 3:**
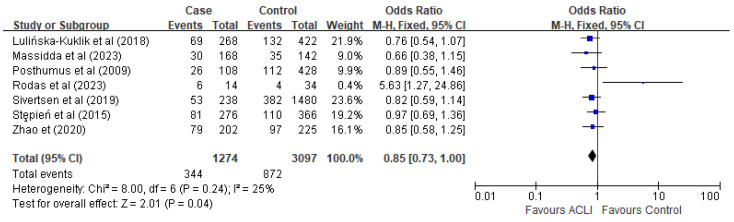
Forest plot of rs 13946 and ACL injury under the allele model (T versus C). The blue points are the study’s OR. The blue horizontal line is 95% CI. References cited Lulińska-Kuklik et al., (2018) [18], Massidda et al., (2023), Posthumus et al., (2009) [13], Rodas et al., (2023) [19], Sivertsen et al., (2019) [26], Stepień-Słodkowska et al., (2015) [27], Zhao et al., (2020) [21]. RevMan 5.4 does not support the *p*-value in italics.

No significant heterogeneity was detected in the analysis of the five genetic models. To evaluate the genetic effect of the rs13946 polymorphism, five genetic models were employed: dominant (TT + TC versus CC), recessive (TT versus TC + CC), homozygote (TT versus CC), heterozygote (TC versus CC), and allelic comparison (T versus C). Our analysis yielded statistically significant findings within the recessive (OR = 1.29, 95% CI [1.06, 1.58], *p* = 0.01) (Figure 2) and allele models (OR = 0.85, 95% CI [0.73, 1.00], *p* = 0.04) (Figure 3). Conversely, the dominant model (OR = 0.95, 95% CI [0.66, 1.37], *p* = 0.79), homozygote model (OR = 0.82, 95% CI [0.56, 1.20], *p* = 0.30), and heterozygote model (OR = 0.91, 95% CI [0.62, 1.33], *p* = 0.61) did not exhibit any significant associations.

**Figure 4:**
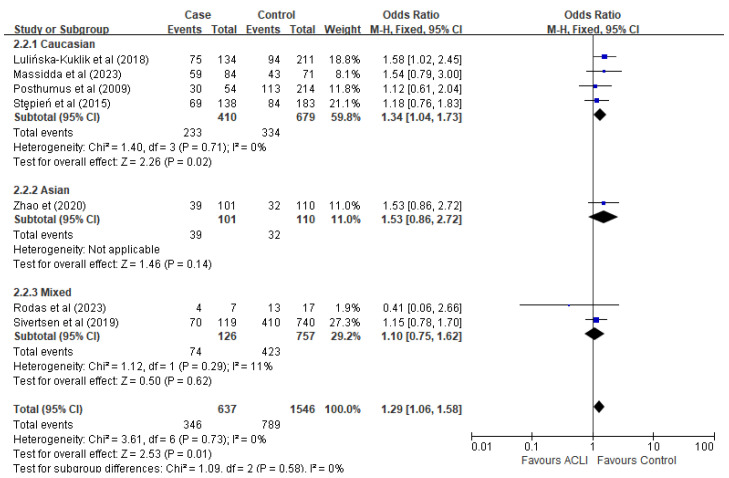
Subgroup-analysis by ethnicity under the recessive model (TT versus TC + CC). The blue points are the study’s OR. The blue horizontal line is 95% CI. References cited Lulińska-Kuklik et al., (2018) [18], Massidda et al., (2023), Posthumus et al., (2009) [13], Stepień-Słodkowska et al., (2015) [27], Zhao et al., (2020) [21], Rodas et al., (2023) [19], Sivertsen et al., (2019) [26]. RevMan 5.4 does not support the *p*-value in italics.

Furthermore, subgroup analyses were undertaken based on ethnicity and gender using the recessive model to explore the potential significant influence of each factor on ACL injury. Of the studies examined, four studies reported the association between ACL injury and rs13946 genetic influence in Caucasians, while three studies were conducted within combined ethnicities, and one study focused on the Asian population; however, the original data showed no association in the Chinese population. Overall, the pooled data showed a significant association in Caucasians (OR = 1.34, 95% CI [1.04, 1.73], *p* = 0.02), whereas no associations were found in mixed population samples (OR = 1.10, 95% CI [0.75, 1.62], *p* = 0.62) (Figure 4).

**Figure 5:**
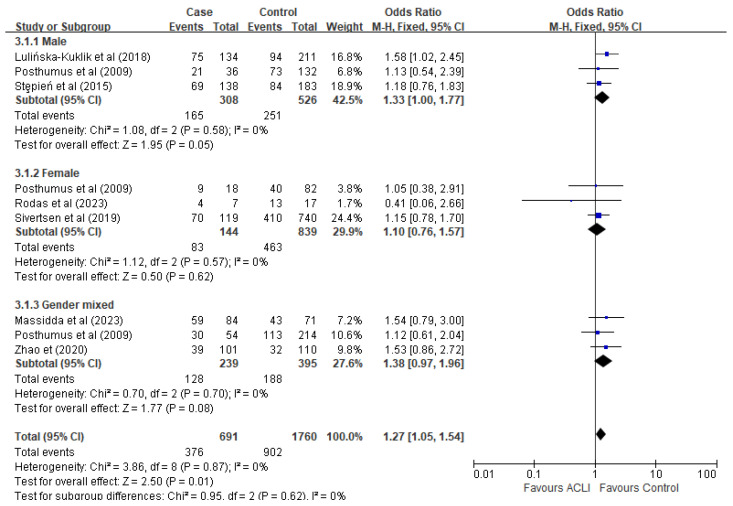
Subgroup-analysis by gender under the recessive model (TT versus TC + CC). The blue points are the study’s OR. The blue horizontal line is 95% CI. References cited Lulińska-Kuklik et al., (2018) [18], Posthumus et al., (2009) [13], Stepień-Słodkowska et al., (2015) [27], Rodas et al., (2023) [19], Sivertsen et al., (2019) [26], Massidda et al., (2023), Zhao et al., (2020) [21]. RevMan 5.4 does not support the *p*-value in italics.

Stratified analysis by gender revealed a marginal significance of the rs13946 polymorphism among male subjects (OR = 1.33, 95% CI [1.00, 1.77], *p* = 0.05) (Figure 5). Conversely, no significant association was identified among female subjects or when genders were combined for analysis.

### 3.5. Sensitivity Analysis and Publication Bias

**Figure 6:**
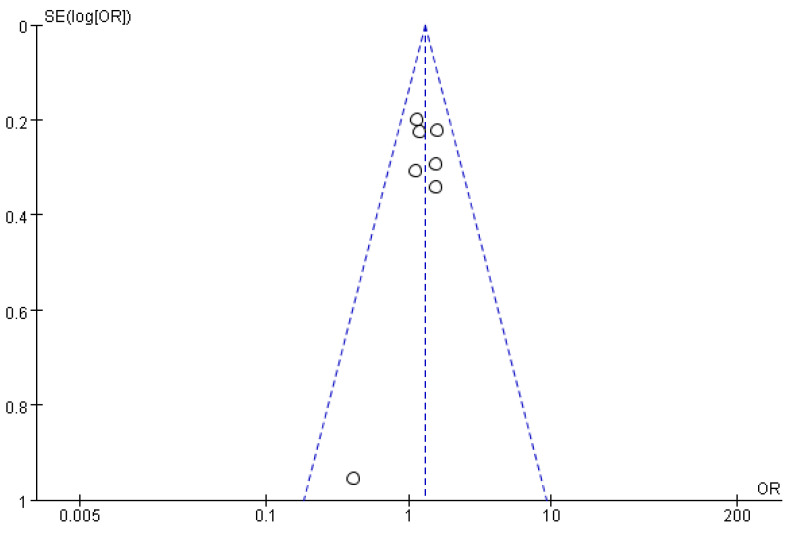
Funnel plot of the association between rs13946 and risk of ACL injury under the recessive model (TT versus TC + CC). The blue dots represent authors cited. The blue dashed lines indicate 95% CI. Vertical dashed line represents the pooled OR. References cited Lulińska-Kuklik et al., (2018) [18], Massidda et al., (2023), Posthumus et al., (2009) [13], Rodas et al., (2023) [19], Sivertsen et al., (2019) [26], Stepień-Słodkowska et al., (2015) [27], Zhao et al., (2020) [21].

After excluding a single study, the pooled OR, 95% confidence interval (CI), and *p* value did not significantly change. Furthermore, the visually symmetrical funnel plot suggests stable results and no significant publication bias among the included studies (Figure 6). The plot is symmetric when publication bias is minimal [31].

## 4. Discussion

The ACL is a critical and complex part of the knee joint, providing essential stability and enabling a wide range of movements. While ACL injuries have long been linked to factors like sports-related trauma and biomechanical influences [32], the exact causes are not fully understood. Research has delved into the genetics governing collagen production, matrix metalloproteinases, interleukins, and cell signaling molecules. Recently, a growing body of evidence suggests that genetic variations play a significant role in ACL ruptures. In this systematic review, seven studies were included with 715 cases, and 1586 healthy individuals were involved in analyzing the association between *COL5A1* rs13946 polymorphism and ACLI. The main finding of this meta-analysis, based on all pooled data, suggests that individuals harboring the TT genotype or T allele of rs13946 exhibit a distinct susceptibility profile concerning ACL injury under the recessive model. Furthermore, stratification analyses by ethnicity and gender reveal that this association was significant in the Caucasian population but not in the Asian and mixed populations. Additionally, the study demonstrates a higher predisposition of the TT genotype among male subjects with ACL injury than females.

The *COL5A1* gene is located on human chromosome 9. It is responsible for encoding a crucial component of type V collagen, a protein vital for the integrity of connective tissues in the body and its impact upon tendon–ligament injury, which is found within the 3′-untranslated region (UTR) [11]. Type V collagen is prevalent in fibril-forming collagens in the cornea, tendon, dermis, bone, and cartilage tissues. Soft tissue injuries in physically active individuals have been associated with alterations in collagen and mutations in genes encoding for these proteins. From a biomechanical perspective, ACL injuries are associated with abrupt deceleration and rapid changes in direction. The cause of ACLI involves a complex interplay between genetic susceptibility and changes in protein structure dynamics, although the precise mechanism underlying this injury remains elusive.

The National Center for Biotechnology Information (NCBI) databases have conducted 13 studies on *COL5A1* rs13946, of which 4 are focused on ACLI research and others are related to soft tissue injury. However, the findings from these studies have shown inconsistencies. In this systematic review, we tried to analyze the association between the polymorphism rs13946 within *COL5A1* in various populations. The results from a meta-analysis of the pooled data showed that *COL5A1* rs13946 and ACLI are significantly associated in the recessive and allele gene models. A significant association was observed only in Caucasians in the recessive model when ethnicity and gender were separated. *COL5A1* and ACLI, however, appear unrelated in most of the included studies when reviewed separately. In component studies by Posthumus et al. [13], Massidda et al. [20], Sivertsen et al. [26], and Zhao et al. [21], the SNP rs13946 did not show significant differences between controls and cases. However, significant associations were demonstrated between the rs13946 haplotype polymorphism and ACL injuries in studies by Lulińska-Kuklik et al. [18], Stȩpień-Słodkowska et al. [27], and Rodas et al. [19]. Rodas et al. [19] found that the C/C genotype, under a recessive model, was more prevalent among female than male footballers. Conversely, Stepień-Słodkowska et al. [27] reported that C/T haplotype polymorphisms were associated with reduced ACL injuries in male athletes.

Despite these mixed findings, our meta-analysis indicates that the TT genotype or T allele, under a recessive model, is associated with an increased risk of ACL injuries, especially in Caucasians. A meta-analysis explored the correlation between *COL5A1* polymorphisms and the susceptibility to tendon–ligament injuries (TLIs) in Caucasian populations. The study identified two SNPs, rs2722 and rs13946, as being significantly associated with TLI, particularly under the homozygous and recessive genetic models compared to the dominant model [33]. These findings are congruent with the results of our meta-analysis. This finding can be better explained by the relatively larger sample sizes and the homogeneity of the ethnic group in studies involving Caucasian populations compared to those involving other populations. The larger sample size enhances the statistical power of the studies, allowing for more robust and reliable detection of associations between *COL5A1* polymorphisms and TLI. Additionally, the genetic homogeneity within the Caucasian group reduces variability and potential confounding factors, leading to more precise and consistent results. These factors contribute to the strength and reliability of the observed associations in Caucasian cohorts.

However, our current analysis yielded a marginal association in males but not females (Figure 5). This finding should be interpreted cautiously, as only three of the seven included studies provided gender-stratified data [13,19,26]. The sample sizes within the female subgroups were small and unevenly distributed. This limited statistical power likely contributed to the absence of a significant effect. Importantly, this finding should not be considered evidence against well-established anatomical [34] and epidemiological data indicating a higher ACL injury risk in females [35]. Instead, it reflects a key limitation in the available literature. It reinforces the need for future studies that include adequately powered, gender-specific analyses to better assess genetic susceptibility in female athletes.

This meta-analysis has several limitations. Only seven experimental studies were included to examine the association between rs13946 and human ACLI. Many of these studies had small sample sizes, increasing the risk of bias and diminishing the statistical power of the analysis. Despite analyzing the data by gender, the small sample size for female participants resulted in findings inconsistent with previous reports. Furthermore, the limited number of studies may not fully represent the genetic diversity of different populations, potentially affecting the generalizability of the results. The inconsistency with earlier studies highlights the need for future research to involve larger, well-balanced, gender-specific cohorts. Such studies would provide more reliable and comprehensive insights into the genetic and physiological factors affecting ACL injuries in both genders, ultimately improving prevention and treatment strategies tailored to men and women.

When studying human genetics, determining an appropriate sample size is inherently challenging. Larger sample sizes generally lead to more robust and reliable findings. However, obtaining a sufficiently large sample within a single study is often difficult. Our previous case–control research included approximately 460 individuals (ACL patients and controls) [36]. Age, training level, and BMI limited our ability to recruit more participants, highlighting a common challenge in genetic studies.

While collecting large datasets is crucial, it is often more practical for researchers to build this incrementally. By contributing data over time through multiple studies, the collective data pool will continue to grow, enabling more comprehensive analyses. We recommend prioritizing the inclusion of diverse populations in initial studies to ensure the broad applicability of findings. As the database expands with contributions from various papers, it will provide a stronger foundation for future investigations to clarify the underlying relationships between ACLI and genetic factors. This collaborative approach is essential for advancing personalized medicine and targeted prevention strategies.

Conversely, our meta-analysis provides clear evidence that the C/- genotype of rs13946 is associated with a reduced risk of ACLI in Caucasians. Future research should explore how the C/- genotype protects against ACLI and determine whether similar advantages are seen in other populations.

## 5. Conclusions

This study supports the association between *COL5A1* rs13946 and the risk of ACL injury, particularly in Caucasians. More specifically, the C/- genotype of rs13946 protects against ACLI in Caucasians. Further research with larger sample sizes and well-balanced gender-specific cohorts is needed to validate this association and draw more definitive conclusions.

## Figures and Tables

**Table 1:** The main characteristics and quality scores of the studies included.

Study	Country	Ethnicity	Sample Size (ACLI/ Control)	Sample	Gender (F/M)	Genotyping Method	Matching	Type of Sports	Diagnosis	Injury Type
Posthumus et al., 2009 [13]	South Africa	Caucasian	129/216	blood	122/223	RFLP	age, sex, height, injury	non-contact, contact exercise	surgery	non-contact, contact
Lulińska-Kuklik et al., 2018 [18]	Poland	Caucasian	134/211	buccal cell	0/345	PCR	age, training volume	football soccer	surgery	non-contact
Rodas et al., 2023 [19]	Spain	mixed Caucasian/African/Latin America	8/38	blood	24/22	PCR	NR	football	medical	non-contact
Massidda et al., 2023 [20]	Italy	Caucasian	86/96	buccal cell	81/101	PCR RFLP	age, training volume	team sport (basketball, football, etc.)	surgery	non-contact
Zhao et al., 2020 [21]	China	Asian	101/110	blood	69/142	PCR RFLP	age, sex	NR	AE	non-contact
Sivertsen et al., 2019 [26]	Norway Finland	Mixed Norwegian Finnish	88/481 31/251	blood	851/0	PCR	type of sport, country	team sport (basketball, football, etc.)	physician MRI and AE	non-contact
Stȩpień-Słodkowska et al., 2015 [27]	Poland	Caucasian	138/183	buccal cell	0/321	RT-PCR	age, training volume	skiing	surgery	non-contact

MRI, magnetic resonance imaging; AE, arthroscopic examination; NR, not reported.

**Table 2:** Genotype frequencies of the rs13946 polymorphism in studies included in the meta-analysis.

Study	ACLI			Alleles of ACLI		Control			Alleles of Control		HWE
	CC	CT	TT	C	CC	CT	TT	C	CC	CT	
Posthumus et al., 2009 [13]	2	22	30	26	82	11	90	113	112	256	>0.05
Lulińska-Kuklik et al., 2018 [18]	10	49	75	69	130	15	102	94	132	158	0.071
Rodas et al., 2023 [19]	3	0	4	6	8	0	4	13	4	30	1
Massidda et al., 2023 [20]	5	20	59	30	138	7	21	43	35	107	0.203
Zhao et al., 2020 [21]	17	45	39	79	123	19	59	3	92	128	>0.05
Sivertsen et al., 2019 [26]	4	45	70	53	185	65	278	410	382	1098	>0.05
Stepień-Słodkowska et al., 2015 [27]	12	57	69	80	196	11	88	84	110	256	0.077

**Table 3:** Risk of bias assessed by the Newcastle–Ottawa Scale.

Study	Newcastle–Ottawa Scale Score				
	Selection	Comparability	Exposure	Total	Design of the Case
Posthumus et al., 2009 [13]	●●○●	●●	●●●	8	case–control study
Lulińska-Kuklik et al., 2018 [18]	●●●●	●●	●●●	9	case–control study
Rodas et al., 2023 [19]	●○●●	○●	●●●	7	cohort study
Massidda et al., 2023 [20]	●●●●	●○	●●●	8	case–control study
Zhao et al., 2020 [21]	●○○●	●○	●●●	6	cross-sectional study
Sivertsen et al., 2019 [26]	●●●●	●○	●●●	8	cohort study
Stepień-Słodkowska et al., 2015 [27]	●●●●	●●	●●●	9	case–control study

The black circle represents one point received. The hollow circle represents one not received.

## Data Availability

Data are available from the corresponding author upon request.

## Supplementary Materials

The following Supporting Information can be downloaded at https://www.mdpi.com/article/10.3390/ijms26136340/s1.
