# Supplementary material for: COL5A1 rs13946 Polymorphism and Anterior Cruciate Ligament Injury: Systematic Review and Meta-Analysis
Source: Int J Mol Sci. 2025 Jun 30;26(13):6340. doi: 10.3390/ijms26136340 (PMC12249684; doi:10.3390/ijms26136340)
Supplement: Supplementary file 1 [file ijms-26-06340-s001.zip › ijms-3640116-supplementary.pdf]

Search strategy

PUBMED: 26

Search number Query Sort By Filters Search Details Results Time

3 (((COL5A1) OR (rs13946)) OR (COL5A1rs13946)) AND (((((((ACL injury) OR (ACL injuries)) OR (ACL rupture)) OR (anterior cruciate ligament injuries)) OR (anterior cruciate ligament injury)) OR (anterior cruciate ligament injuries)) OR (anterior cruciate ligament ruptures))
Most Recent ("COL5A1"[All Fields] OR "rs13946"[All Fields]) AND ("anterior cruciate ligament injuries"[MeSH Terms] OR ("anterior"[All Fields] AND "cruciate"[All Fields] AND "ligament"[All Fields] AND "injuries"[All Fields]) OR "anterior cruciate ligament injuries"[All Fields] OR ("acl"[All Fields] AND "injury"[All Fields]) OR "acl injury"[All Fields] OR ("anterior cruciate ligament injuries"[MeSH Terms] OR ("anterior"[All Fields] AND "cruciate"[All Fields] AND "ligament"[All Fields] AND "injuries"[All Fields]) OR "anterior cruciate ligament injuries"[All Fields] OR ("acl"[All Fields] AND "injuries"[All Fields]) OR "acl injuries"[All Fields]) OR (("anterior cruciate ligament"[MeSH Terms] OR ("anterior"[All Fields] AND "cruciate"[All Fields] AND "ligament"[All Fields]) OR "anterior cruciate ligament"[All Fields] OR "acl"[All Fields]) AND ("ruptur"[All Fields] OR "rupture"[MeSH Terms] OR "rupture"[All Fields] OR "ruptured"[All Fields] OR "ruptures"[All Fields] OR "rupturing"[All Fields])) OR ("anterior cruciate ligament injuries"[MeSH Terms] OR ("anterior"[All Fields] AND "cruciate"[All Fields] AND "ligament"[All Fields] AND "injuries"[All Fields]) OR "anterior cruciate ligament injuries"[All Fields]) OR ("anterior cruciate ligament injuries"[MeSH Terms] OR ("anterior"[All Fields] AND "cruciate"[All Fields] AND "ligament"[All Fields] AND "injuries"[All Fields]) OR "anterior cruciate ligament injuries"[All Fields] OR ("anterior"[All Fields] AND "cruciate"[All Fields] AND "ligament"[All Fields] AND "injury"[All Fields]) OR "anterior cruciate ligament injury"[All Fields]) OR ("anterior cruciate ligament injuries"[MeSH Terms] OR ("anterior"[All Fields] AND "cruciate"[All Fields] AND "ligament"[All Fields] AND "injuries"[All Fields]) OR "anterior cruciate ligament injuries"[All Fields]) OR (("anterior cruciate ligament"[MeSH Terms] OR ("anterior"[All Fields] AND "cruciate"[All Fields] AND "ligament"[All Fields]) OR "anterior cruciate ligament"[All Fields]) AND ("ruptur"[All Fields] OR "rupture"[MeSH Terms] OR "rupture"[All Fields] OR "ruptured"[All Fields] OR "ruptures"[All Fields] OR "rupturing"[All Fields]))) **26** 0:01:47
2. ((((((ACL injury) OR (ACL injuries)) OR (ACL rupture)) OR (anterior cruciate ligament injuries)) OR (anterior cruciate ligament injury)) OR (anterior cruciate ligament injuries)) OR (anterior cruciate ligament ruptures)
Most Recent "anterior cruciate ligament injuries"[MeSH Terms] OR ("anterior"[All Fields] AND "cruciate"[All Fields] AND "ligament"[All Fields] AND "injuries"[All Fields]) OR "anterior cruciate ligament injuries"[All Fields] OR ("acl"[All Fields] AND "injury"[All Fields]) OR "acl injury"[All Fields] OR ("anterior cruciate ligament injuries"[MeSH Terms] OR ("anterior"[All Fields] AND "cruciate"[All Fields] AND "ligament"[All Fields] AND "injuries"[All Fields]) OR "anterior cruciate ligament injuries"[All Fields] OR ("acl"[All Fields] AND "injuries"[All Fields]) OR "acl injuries"[All Fields]) OR (("anterior cruciate ligament"[MeSH Terms] OR ("anterior"[All Fields] AND "cruciate"[All Fields] AND "ligament"[All Fields]) OR "anterior cruciate ligament"[All Fields] OR "acl"[All Fields]) AND ("ruptur"[All Fields] OR "rupture"[MeSH Terms] OR "rupture"[All Fields] OR "ruptured"[All Fields] OR "ruptures"[All Fields] OR "rupturing"[All Fields])) OR ("anterior cruciate ligament injuries"[MeSH Terms] OR ("anterior"[All Fields] AND "cruciate"[All Fields] AND "ligament"[All Fields] AND "injuries"[All Fields]) OR "anterior cruciate ligament injuries"[All Fields]) OR ("anterior cruciate ligament injuries"[MeSH Terms] OR ("anterior"[All Fields] AND "cruciate"[All Fields] AND "ligament"[All Fields] AND "injuries"[All Fields]) OR "anterior cruciate ligament injuries"[All Fields] OR

("anterior"[All Fields] AND "cruciate"[All Fields] AND "ligament"[All Fields] AND "injury"[All Fields]) OR "anterior cruciate ligament injury"[All Fields]) OR ("anterior cruciate ligament injuries"[MeSH Terms] OR ("anterior"[All Fields] AND "cruciate"[All Fields] AND "ligament"[All Fields] AND "injuries"[All Fields]) OR "anterior cruciate ligament injuries"[All Fields]) OR (("anterior cruciate ligament"[MeSH Terms] OR ("anterior"[All Fields] AND "cruciate"[All Fields] AND "ligament"[All Fields]) OR "anterior cruciate ligament"[All Fields]) AND ("ruptur"[All Fields] OR "rupture"[MeSH Terms] OR "rupture"[All Fields] OR "ruptured"[All Fields] OR "ruptures"[All Fields] OR "rupturing"[All Fields])) **22,286** 0:01:29

1 ((COL5A1) OR (rs13946)) OR (COL5A1rs13946)

Most Recent "COL5A1"[All Fields] OR "rs13946"[All Fields] **602** 23:58:33

SCOPUS

Set search alert

Advanced query
((TITLE-ABS-KEY (anterior AND cruciate AND ligament AND injury) OR TITLE-ABS-KEY (anterior AND cruciate AND ligament AND injuries) OR TITLE-ABS-KEY (acl AND injury) OR TITLE-ABS-KEY (acl AND injuries) OR TITLE-ABS-KEY (acl AND tears) OR TITLE-ABS-KEY (acl AND tear) OR TITLE-ABS-KEY (acl AND rupture) OR TITLE-ABS-KEY (acl AND ruptures))) AND ((TITLE-ABS-KEY (col5a1) OR TITLE-ABS-KEY (col5a1 AND rs13946) OR TITLE-ABS-KEY (rs13946)))

Search History

3. ((TITLE-ABS-KEY (anterior AND cruciate AND ligament AND injury) OR TITLE-ABS-KEY (anterior AN D cruciate AND ligament AND injuries) OR TITLE-ABS-KEY (acl AND injury) OR TITLE-ABS-KEY (acl A ND injuries) OR TITLE-ABS-KEY (acl AND tears) OR TITLE-ABS-KEY (acl AND tear) OR TITLE-ABS-K EY (acl AND rupture) OR TITLE-ABS-KEY (acl AND ruptures))) AND ((TITLE-ABS-KEY (col5a1) OR T ITLE-ABS-KEY (col5a1 AND rs13946) OR TITLE-ABS-KEY(rs13946))) **30 results**

2. (TITLE-ABS-KEY (col5a1) OR TITLE-ABS-KEY (col5a1 AND rs13946) OR TITLE-ABS-KEY (rs13946)) **735 results**

1. (TITLE-ABS-KEY (anterior AND cruciate AND ligament AND injury) OR TITLE-ABS-KEY (anterior AN D cruciate AND ligament AND injuries) OR TITLE-ABS-KEY (acl AND injury) OR TITLE-ABS-KEY (acl A ND injuries) OR TITLE-ABS-KEY (acl AND tears) OR TITLE-ABS-KEY (acl AND tear) OR TITLE-ABS-K EY (acl AND rupture) OR TITLE-ABS-KEY (acl AND ruptures)) **27,324 results**

Web of Science Search Strategy

Entitlements	#	Search Query	Database	Results	Date Run
"- WOS.IC: 1993 to 2024<br>- WOS.CCR: 1985 to 2024<br>- WOS.SCI: 1975 to 2024<br>- WOS.AHCI: 1975 to 2024<br>- WOS.BHCI: 2005 to 2024<br>- WOS.BSCI: 2005 to 2024<br>- WOS.ESCI: 2019 to 2024<br>- WOS.ISTP: 1990 to 2024<br>- WOS.SSCI: 1965 to 2024<br>- WOS.ISSHP: 1990 to 2024"	1	"(((((((ALL=(Anterior cruciate ligament injuries)) OR ALL=(Anterior cruciate ligament injury)) OR ALL=(Anterior cruciate ligament rupture)) OR ALL=(Anterior cruciate ligament ruptures)) OR ALL=(ACL injury)) OR ALL=(ACL injuries)) OR ALL=(ACL rupture)) OR ALL=(ACL ruptures) "	Web of Science Core Collection	**18311**	Tue Mar 26 2024 13:27:21 GMT+0800 (China Standard Time)
"- WOS.IC: 1993 to 2024<br>- WOS.CCR: 1985 to 2024<br>- WOS.SCI: 1975 to 2024<br>- WOS.AHCI: 1975 to 2024<br>- WOS.BHCI: 2005 to 2024<br>- WOS.BSCI: 2005 to 2024<br>- WOS.ESCI: 2019 to 2024<br>- WOS.ISTP: 1990 to 2024<br>- WOS.SSCI: 1965 to 2024<br>- WOS.ISSHP: 1990 to 2024"	2	"((ALL=(COL5A1)) OR ALL=(rs13946)) OR ALL=(COL5A1 rs13946)"	Web of Science Core Collection	**707**	Tue Mar 26 2024 13:28:29 GMT+0800 (China Standard Time)
"- WOS.IC: 1993 to 2024<br>- WOS.CCR: 1985 to 2024<br>- WOS.SCI: 1975 to 2024<br>- WOS.AHCI: 1975 to 2024<br>- WOS.BHCI: 2005 to 2024<br>- WOS.BSCI: 2005 to 2024<br>- WOS.ESCI: 2019 to 2024<br>- WOS.ISTP: 1990 to 2024<br>- WOS.SSCI: 1965 to 2024<br>- WOS.ISSHP: 1990 to 2024"	3	"#1 AND #2 "	Web of Science Core Collection	**64**	Tue Mar 26 2024 13:28:44 GMT+0800 (China Standard Time)

CNKI

1. Qianjiaocharendai+qianjiaocharendaisunshang AND COL5A1+COL5ALjiyin **Results:21**

Ebscohost Thursday, April 11, 2024 2:57:38 AM

#	Query	Limiters/Expanders	Last Run Via	Results
S3	(TX col5a1 OR rs13946) AND (S1 AND S2)	Expanders - Apply equivalent subjects<br>Search modes - Find all my search terms	Interface - EBSCOhost Research Databases<br>Search Screen - Advanced Search<br>Database - MEDLINE	4
S2	TX col5a1 OR rs13946	Limiters - Human; Available in Library Collection<br>Expanders - Apply equivalent subjects<br>Search modes - Find all my search terms	Interface - EBSCOhost Research Databases<br>Search Screen - Advanced Search<br>Database - MEDLINE	88
S1	TX (acl injury or anterior cruciate ligament injury or acl tear or anterior cruciate ligament tear or acl rupture)	Limiters - Human; Available in Library Collection<br>Expanders - Apply equivalent subjects<br>Search modes - Find all my search terms	Interface - EBSCOhost Research Databases<br>Search Screen - Advanced Search<br>Database - MEDLINE	1138
